# Supplementary figures and images for: Abnormal LAMP1 glycosylation may play a role in Niemann-Pick disease, type C pathology
Source: PLoS One. 2020 Jan 30;15(1):e0227829. doi: 10.1371/journal.pone.0227829 (PMC6992233; doi:10.1371/journal.pone.0227829)

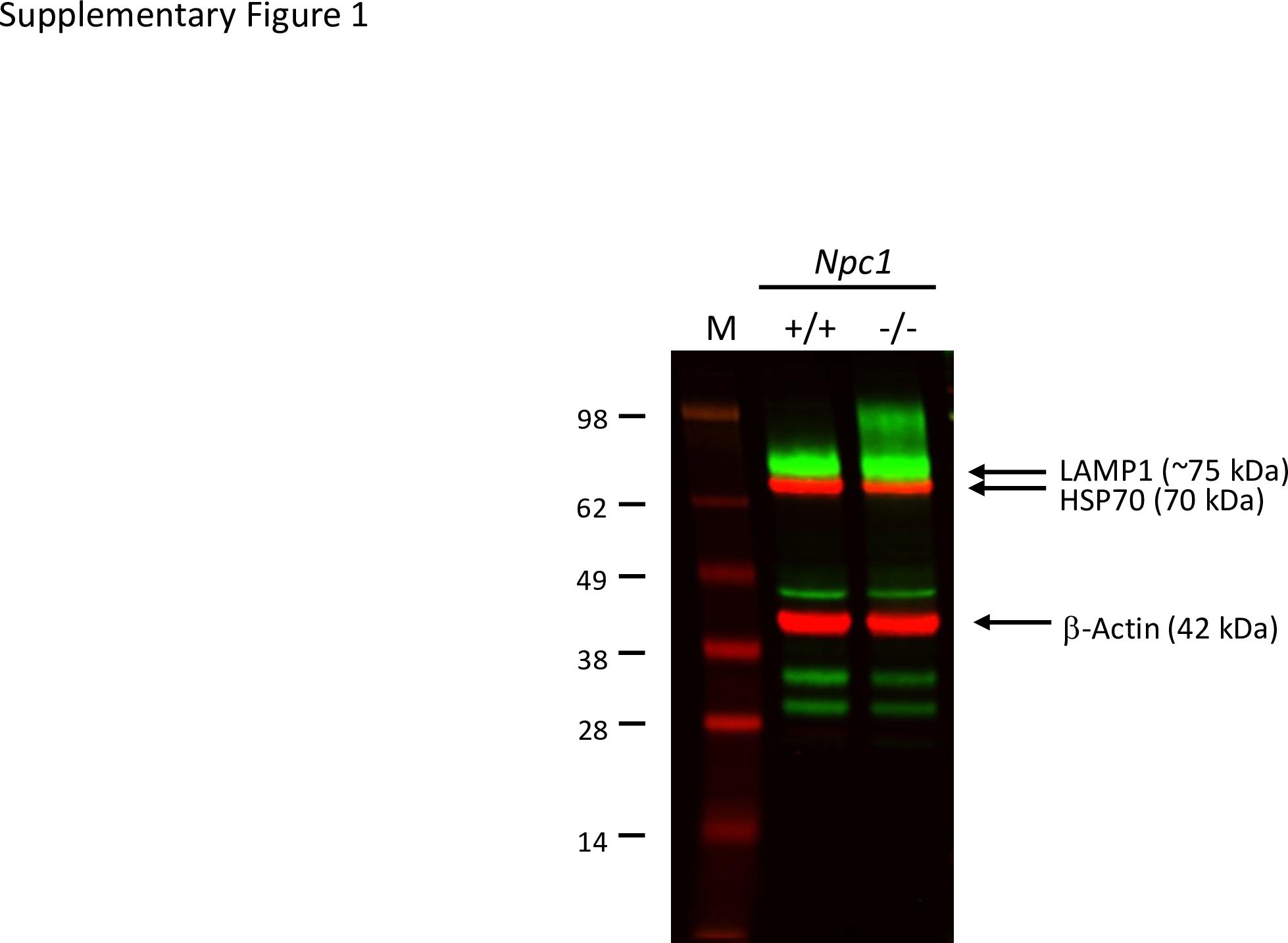

Supplement: S1 Fig — (A) Western blot of total cerebellar extract of Npc1+/+ and Npc1−/− mice for LAMP1 (green) and heat shock protein 70 (HSP70) (upper red). Actin was used as a loading control. (TIF) [file pone.0227829.s001.tif]

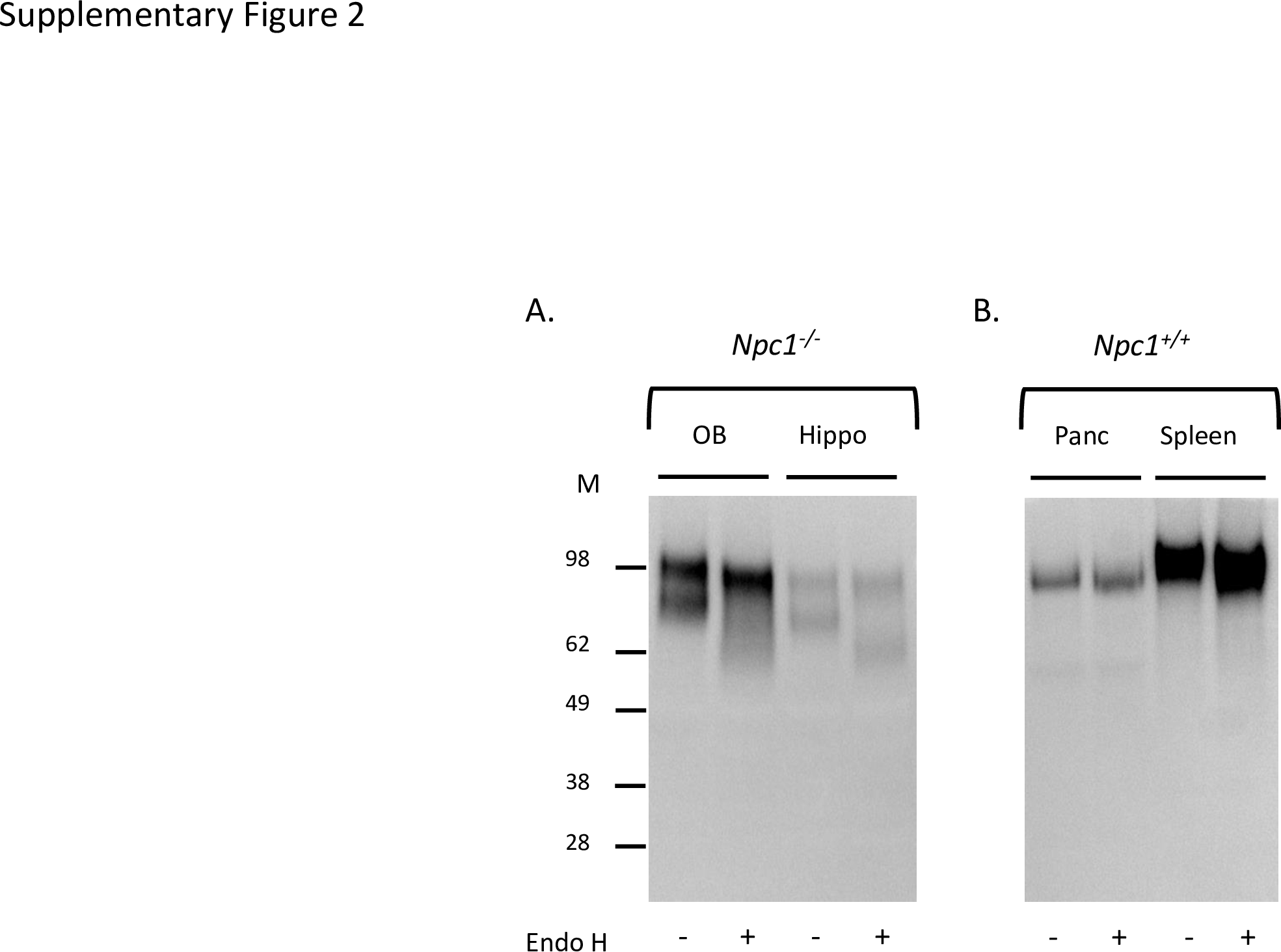

Supplement: S2 Fig — (A) LAMP1 Western blot of 9-week Npc1−/− olfactory bulb (OB) and hippocampus (Hippo) lysate, treated with (+) and without (-) Endo H. (B) LAMP1 Western blot of 9-week Npc1+/+ pancreas (Panc) and spleen lysate, treated with (+) and without (-) Endo H. (TIF) [file pone.0227829.s002.tif]

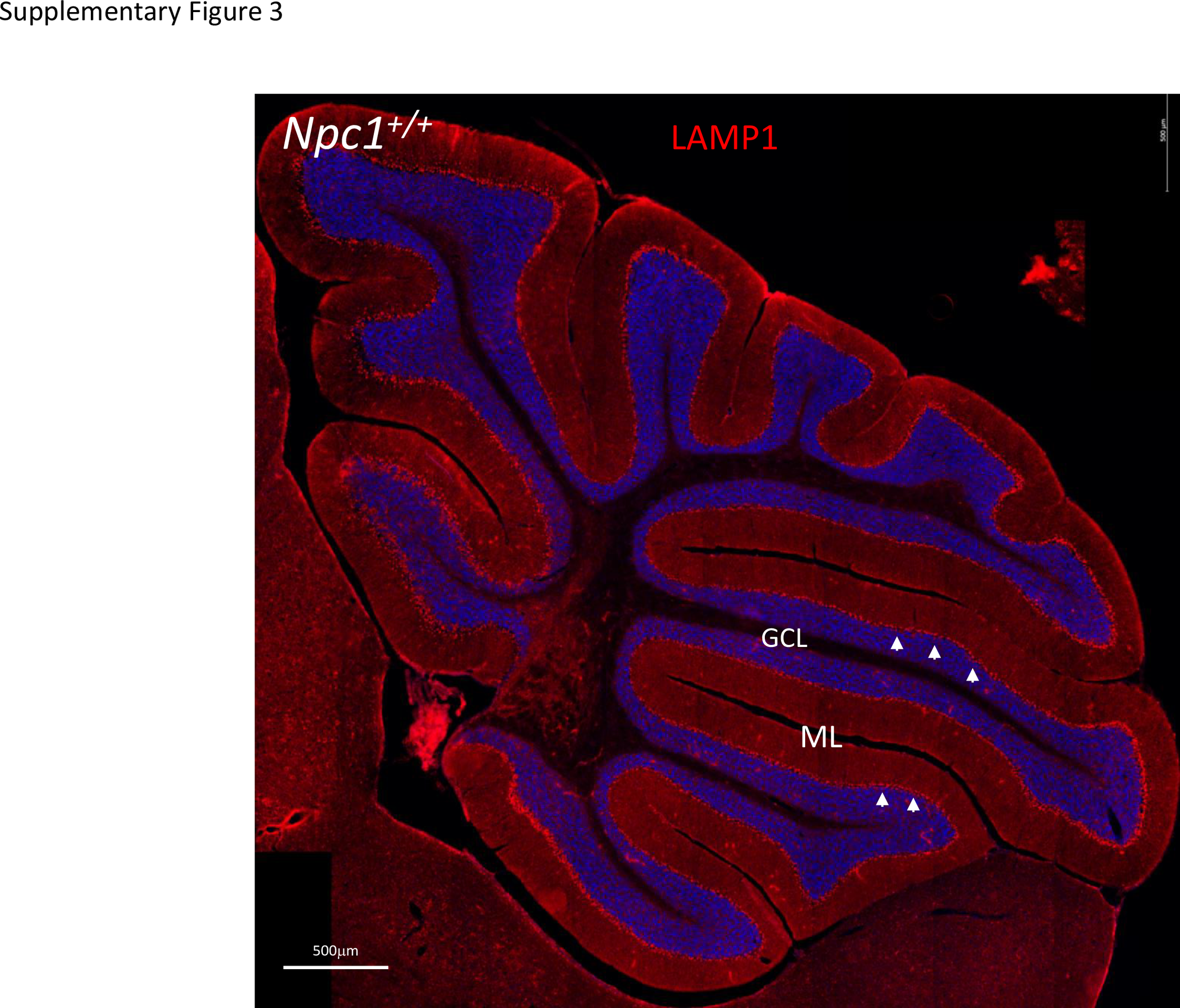

Supplement: S3 Fig — Low magnification image of the whole cerebellar section stained with LAMP1 (red). Arrowheads indicate positive staining of the Purkinje cell layer. Granule cell layer (GCL); Molecular layer (ML). (TIF) [file pone.0227829.s003.tif]

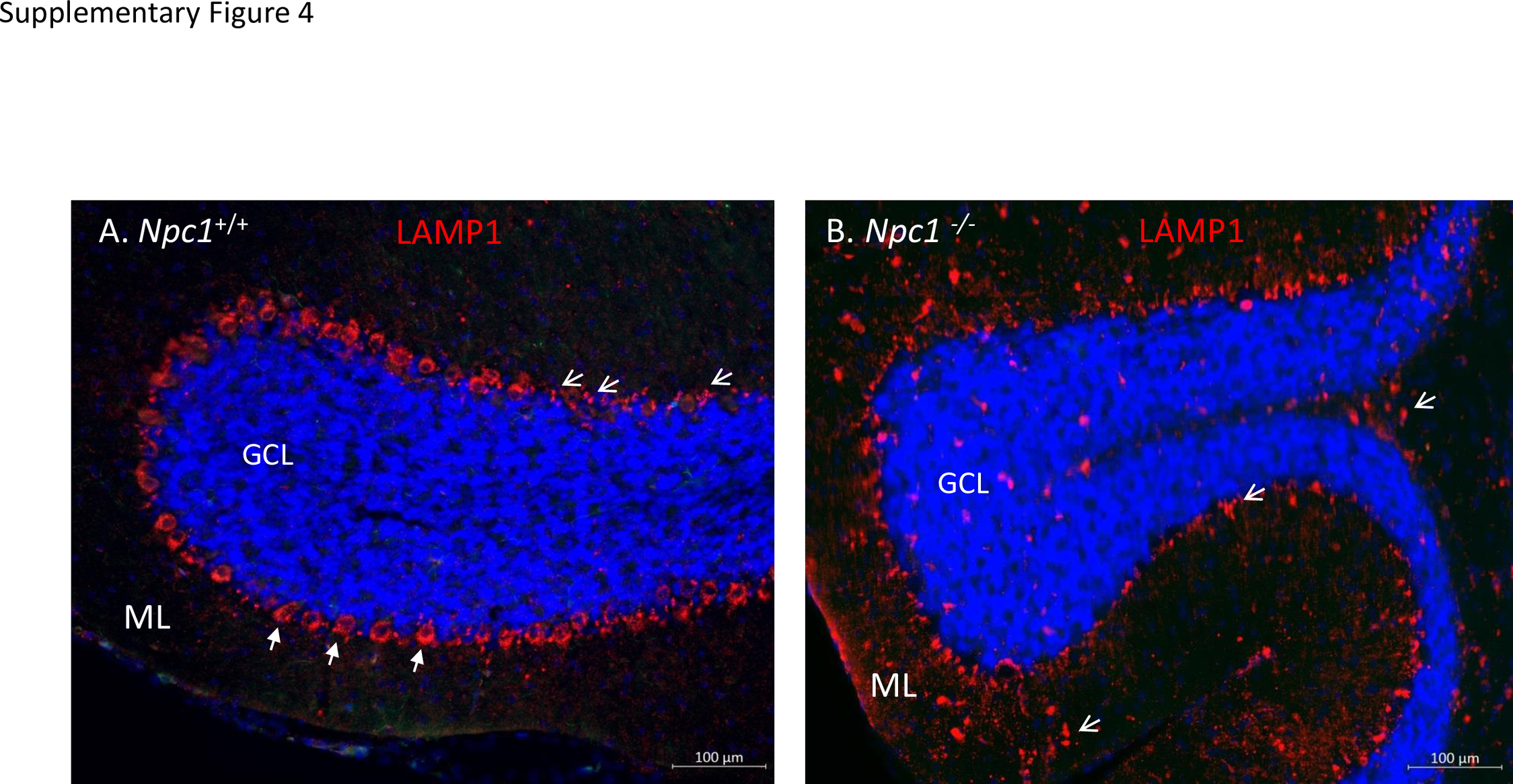

Supplement: S4 Fig — Higher magnification image of a lobule of Npc1+/+ (A) and Npc1-/- (B) stained with LAMP1 (red). A. Arrows indicate positive LAMP1 staining of the Purkinje cell layer (closed arrows: Purkinje neuron soma; open arrows: other LAMP1 positive cells). B. LAMP1 staining is more disordered in the Purkinje cell layer of Npc1-/- cerebellum and has strong staining in the molecular layer. Granule cell layer (GCL); Molecular layer (ML). (TIF) [file pone.0227829.s004.tif]

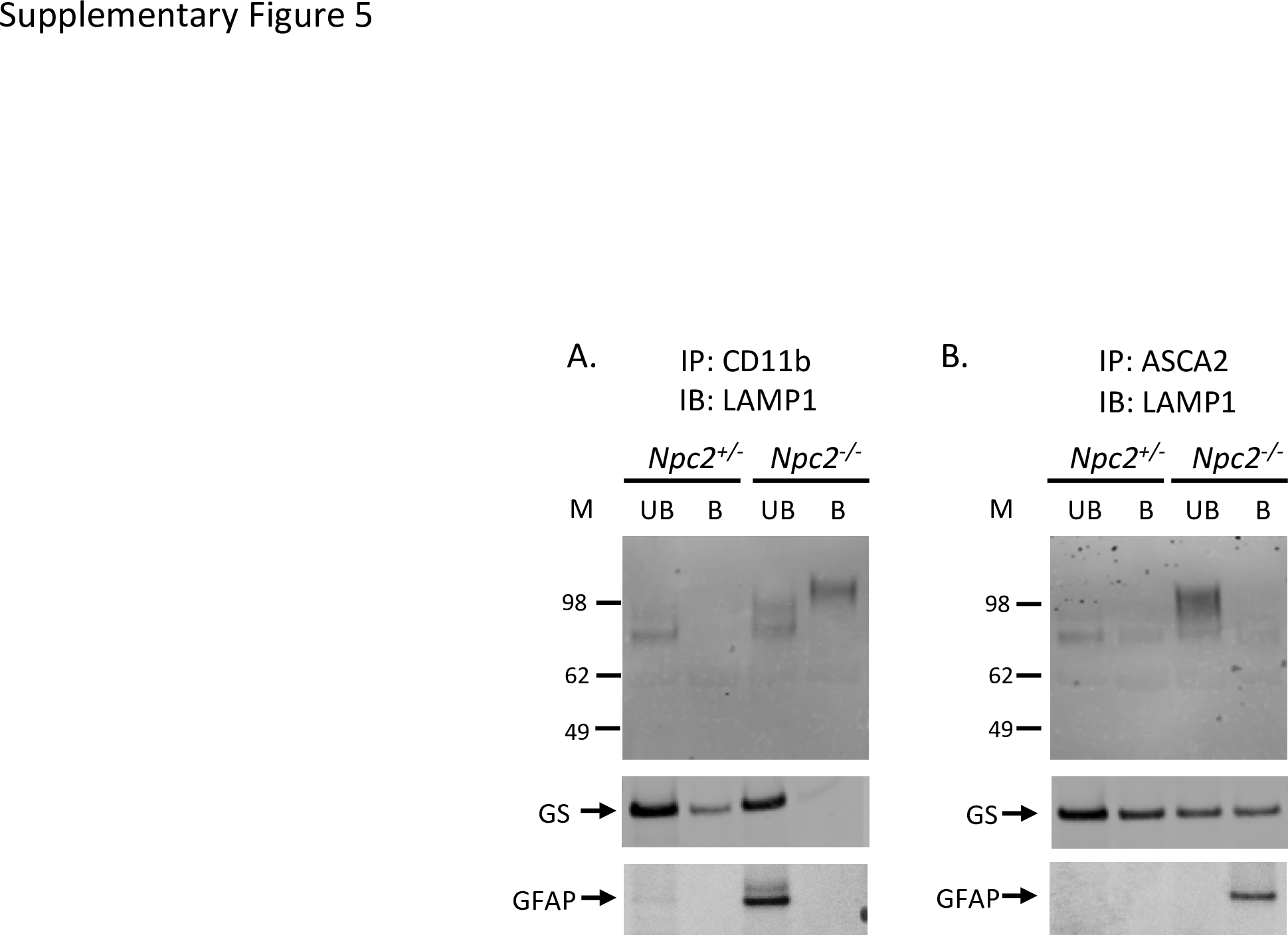

Supplement: S5 Fig — (A & B) LAMP1 Western blot of cell lysates from 16-week-old Npc2+/- and Npc2−/− mouse cerebella that underwent tissue dissociation and subsequent CD11b (microglia) or ASCA-2 (astrocyte) immunoprecipitation. (A) Lanes show the un-bound (UB) and bound (B) fractions of CD11b immunoprecipitation. (B) Lanes show the un-bound (UB) and bound (B) fractions of ASCA2 immunoprecipitation. Glutamine synthase (GS) and Glial fibrillary acid protein (GFAP). (TIF) [file pone.0227829.s005.tif]

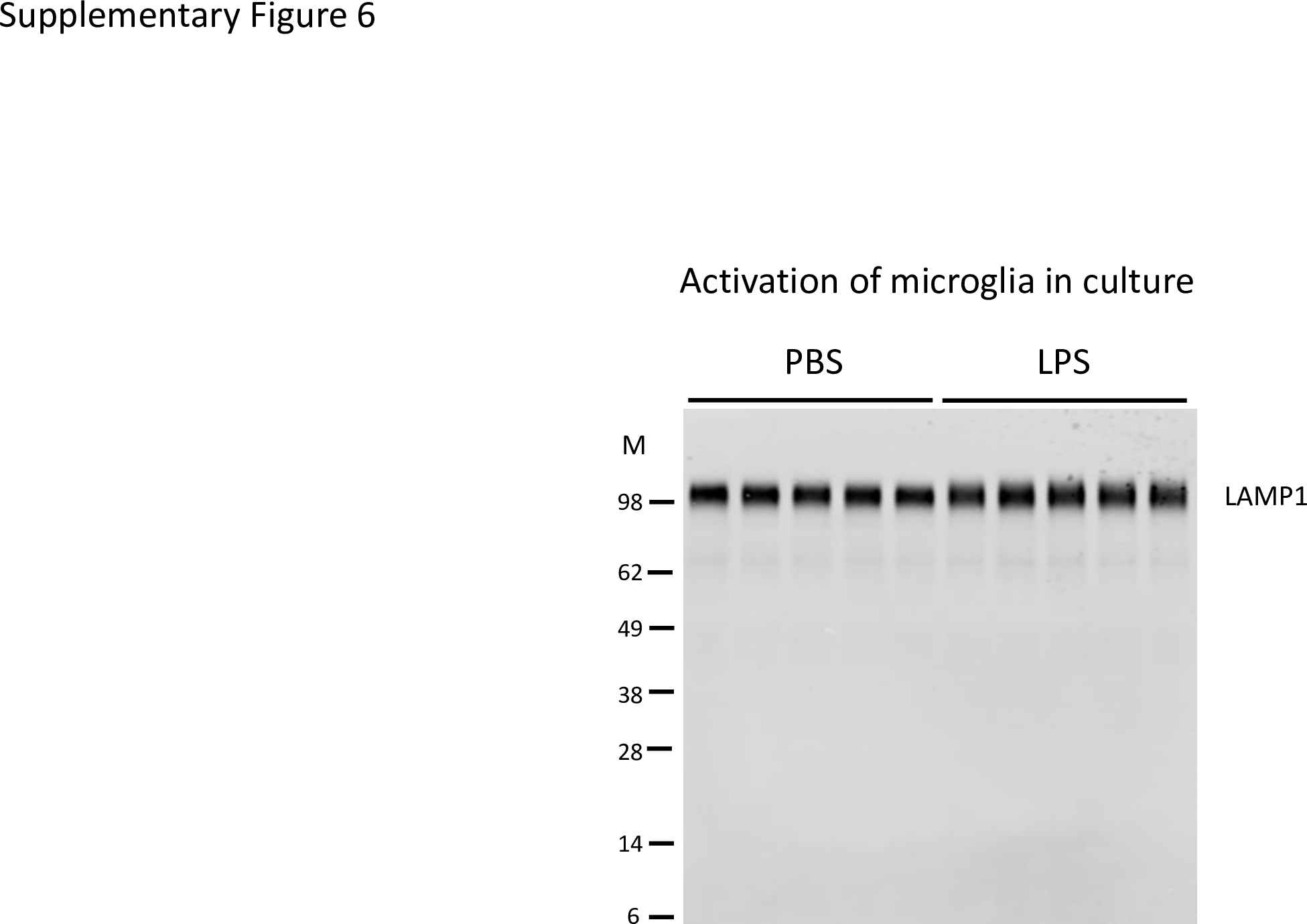

Supplement: S6 Fig — LAMP1 Western blot of microglia cell lysate treated with LPS or PBS (vehicle control). (TIF) [file pone.0227829.s006.tif]

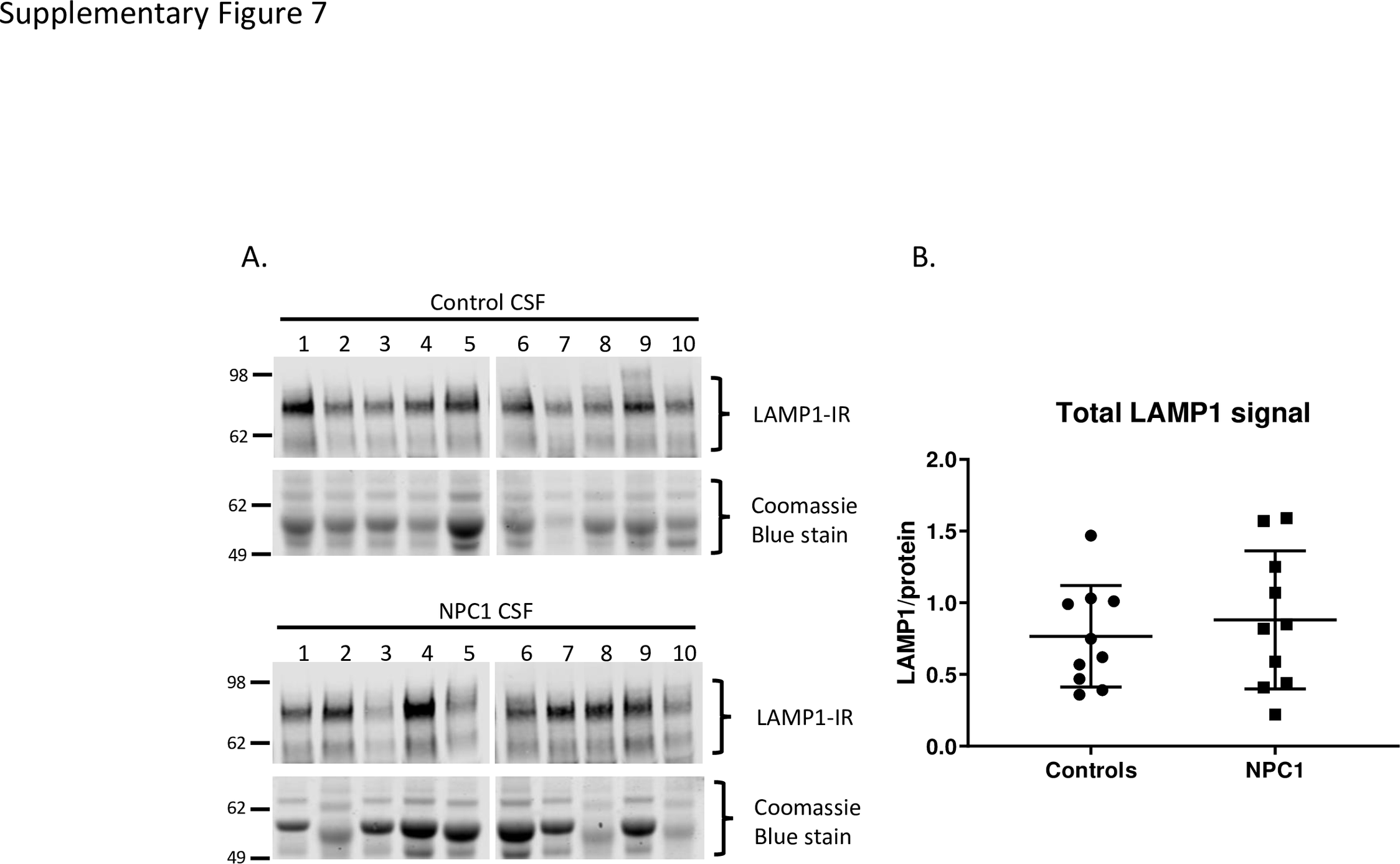

Supplement: S7 Fig — A. Western blot of LAMP1 in CSF of NPC1 patient (lower panel) and adult healthy controls (upper panel). Coomassie Blue stain (indicated) was used to normalize LAMP1 to total protein. The LAMP1 band intensity was quantified and standardized to the total protein signal in the whole respective lane. B. The LAMP1 total protein ratios (mean +/- SD) for NPC1 patients and healthy controls is graphed to the right of the Western blots. (TIF) [file pone.0227829.s007.tif]
